# Chemical Composition and Antimicrobial Properties of *Mentha* × *piperita* cv. ‘Kristinka’ Essential Oil

**DOI:** 10.3390/plants10081567

**Published:** 2021-07-30

**Authors:** Ippolito Camele, Daniela Gruľová, Hazem S. Elshafie

**Affiliations:** 1 School of Agricultural, Forestry, Food and Environmental Sciences (SAFE), University of Basilicata, Viale dell’Ateneo Lucano 10, 85100 Potenza, Italy; ippolito.camele@unibas.it; 2 Department of Ecology, Faculty of Humanities and Natural Sciences, University of Prešov, 17. Novembra 1, 08001 Prešov, Slovakia; daniela.grulova@unipo.sk

**Keywords:** medicinal plants, GC-MS, postharvest diseases, biological control, cell membrane permeability

## Abstract

Several economically important crops, fruits and vegetables are susceptible to infection by pathogenic fungi and/or bacteria postharvest or in field. Recently, plant essential oils (EOs) extracted from different medicinal and officinal plants have had promising antimicrobial effects against phytopathogens. In the present study, the potential microbicide activity of *Mentha* × *piperita* cv. ‘Kristinka’ (peppermint) EO and its main constituents have been evaluated against some common phytopathogens. In addition, the cell membrane permeability of the tested fungi and the minimum fungicidal concentrations were measured. The antifungal activity was tested against the following postharvest fungi: *Botrytis cinerea*, *Monilinia fructicola*, *Penicillium expansum* and *Aspergillus niger*, whereas antibacterial activity was evaluated against *Clavibacter michiganensis*, *Xanthomonas campestris*, *Pseudomonas savastanoi* and *P. syringae* pv. *phaseolicola*. The chemical analysis has been carried out using GC-MS and the main components were identified as menthol (70.08%) and menthone (14.49%) followed by limonene (4.32%), menthyl acetate (3.76%) and β-caryophyllene (2.96%). The results show that the tested EO has promising antifungal activity against all tested fungi, whereas they demonstrated only a moderate antibacterial effect against some of the tested bacteria.

## 1. Introduction

Many microorganisms cause different plant diseases in field and/or postharvest. Without proper treatments, they can cause losses or decrease the shelf life of fruits and vegetables [1,2]. Although synthetic pesticides efficiently control diseases, their application is restricted, particularly postharvest, because of consumer concern for human health conditions, the harmful effects on the environment and the development of new resistant strains [3,4,5].

There are strict regulations worldwide regarding the minimum pesticide residue levels in the edible portion of the fresh vegetable and fruits for protecting human health and the environment [1,5]. On the other hand, in Europe, synthetic fungicides are prohibited in postharvest applications. For that reason, the discovery of new natural substances, such as plant essential oils (EOs), for controlling phytopathogens, especially in postharvest conditions, has attracted great interest recently. Several research projects reported the antifungal efficacy of plant EOs against postharvest fruit pathogens, being considered natural, safe and biodegradable alternatives [6,7,8,9]. 

*Mentha* × *piperita* L. (peppermint) is a perennial plant that is widespread throughout the Mediterranean region [10]. Peppermint, a plant in the Family *Lamiaceae*, has long been considered an economically important [11]. It was already known in Egyptian, Greek and Roman medicine for its wide benefits for human health, especially for digestive and diuretic problems and as a remedy for coughs and colds [12]. Peppermint has several medicinal uses such as treating stomach-aches, chest pains and for treating irritable bowel syndrome [13]. Peppermint EO can be extracted from the aerial parts of the flowering plant, from dried leaves or from fresh flowers [11,14]. Many studies reported the chemical composition of peppermint EO, which is composed mainly of menthol, menthone, menthofuran, 1,8-cineole, and menthyl acetate [15]. Previous studies revealed that most peppermint EO is rich in pulegone, menthon, menthol, carvone, 1,8-cineole, limonene and β-caryophyllene [16]. Regarding the chemical composition of peppermint EO, some previous studies have analysed its chemical composition and principal single constituents and found that the respective percentage of different peppermint species varied depending upon the origins of the plant, species as well as the possible variation within the same species [16]. In addition, there are some factors, such as physiological and environmental conditions, genetic and evolution that can also determine the chemical variability of peppermint EO [17].

Bibliographic research revealed that the plant EOs from different species of peppermint possess potential antimicrobial activity against different plant pathogens [18] as well as insecticidal activity against stored product [19]. Several researchers have also reported the promising biological activities of peppermint EO against different phyto- and food pathogens, especially against Gram-positive bacteria such as *Staphylococcus aureus* and *Enterococcus faecalis*, as reported by Jirovetz et al. [11]. Researchers at the University of Prešov bred a variety of peppermint with a very high content of the main constituents—menthol and menthone [20]. 

The current study aims to evaluate the in vitro antimicrobial activity of peppermint EO against some common postharvest fungal pathogens and some pathogenic bacteria. This research aims also to study the mode of antimicrobial actions and the minimum fungicidal concentrations, both for the EO and for its main components.

In particular, the main objectives of the current study were to: (i) identify the main components of the *Mentha* × *piperita* cv. ‘Kristinka’ in the harvest season 2020 cultivated in Prešov, Slovakia; (ii) screen the antifungal effect of the extracted EO against *Monilinia fructicola*, *Aspergillus niger*, *Penicillium expansum* and *Botrytis cinerea*; (iii) evaluate the antibacterial affect against *Clavibacter michiganensis*, *Xanthomonas campestris*, *Pseudomonas savastanoi* and *P. syringae* pv. *phaseolicola*; (iv) study the effect of EO and its two main constituents (menthol an menthone) on the fungal cell membrane permeability (CMP); (v) determine the minimum fungicidal concentration (MFC) of the studied EO and its two main constituents.

## 2. Results

### 2.1. Identification of M. piperita EO Components

Essential oil of *M.* × *piperita* cv. ‘Kristinka’ was hydrodistilled and qualitatively analysed using GC-MS for determining the main components as mentioned below. Average amount of EO was 0.4 ± 0.02% from plant materials. Qualitative parameters are summarised in Table 1. The most principal component identified was menthol (70.08 ± 0.05%), followed by menthone (14.49 ± 0.01%). This is the typical characteristic of the new cultivar Kristinka, where menthol is the dominant component with higher quantity. Other dominant components were limonene (4.32 ± 0.03%), menthyl acetate (3.76 ± 0.01%) and *β*-caryophyllene (2.96 ± 0.04%). Oxygenated monoterpenes presented 89.13% of the identified chemical group. Sesquiterpenes hydrocarbons (5.46%) and monoterpenes hydrocarbons (5.26%) followed with the almost the same quantity. Among the different chemical groups, oxygenated sesquiterpenes, such as spathulenol compound, were also present in very low quantities (0.03%).

### 2.2. Antibacterial Activity

The results of the antibacterial activity assay showed that the positive control (Tetracycline 1.6 mg/mL) demonstrated the highest inhibition against all tested phytopathogenic bacteria (Table 2). However, peppermint EO showed the highest significant antibacterial activity against *P. syringae* pv. *phaseolicola* (the diameter of inhibition zone was 39.5 mm) similar to tetracycline (1.6 mg/mL) where the diameter of its inhibition zone was 40 mm. In addition, there was a moderate activity against *C. michiganensis* at 10 mg/mL and low activity against *P. savastanoi* only at 10 mg/mL. On the other hand, there was no activity against *X. campestris*.

### 2.3. Antifungal Activity

**Figure 1:**
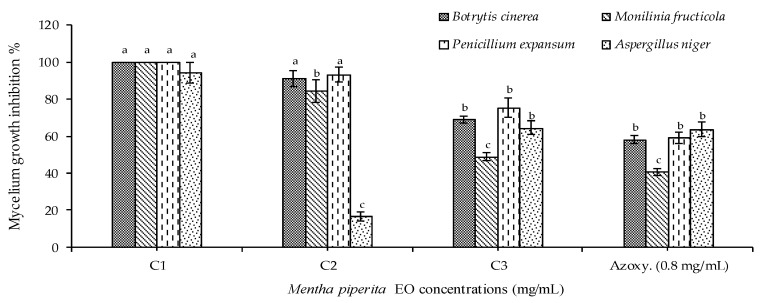
Antifungal activity of *M. piperita* EO. Where: C1: 5.0 mg/mL; C2: 1.0 mg/mL; C3: 0.1 mg/mL. Bars with different letters for each tested fungus indicate mean values significantly different at *p* < 0.05 according to two-way ANOVA combined with Duncan post hoc multiple comparison test.

The results of the fungicidal activity of peppermint EO are presented in Figure 1, where it showed the highest significant inhibition the mycelium growth of *B. cinerea* and *P. expansum* in plates at the two tested concentrations (1 and 5 mg/mL), whereas peppermint EO at 5 mg/mL demonstrated the highest inhibition against *M. fructicola* and *A. niger*. The lowest inhibition effect was observed in the case of the tested concentration 0.1 mg/mL against all tested pathogenic fungi, which was insignificantly different from the positive control (Azoxystrobin, 0.8 µL/mL). In fact, the inhibition of fungal mycelium growth in plates is considered as a general indication of the efficacy of the tested treatments, whereas the MFC assay is considered a more accurate test for determining the lowest concentration required to inhibit the visible growth of the microorganism. 

### 2.4. Fungal Cell Membrane Permeability Assay

This assay was carried out to explain the possible mechanism of the antifungal activity of the tested EO. In general, the fungicidal effect of EO depends on the destruction of the fungal cell membrane that increases the cell permeability. For that reason, the current assay was performed to investigate the effect of mint EO and its main single constituents on the CMP of the tested phytopathogenic fungi by measuring their electric conductivity (EC) [21,22].

**Figure 2:**
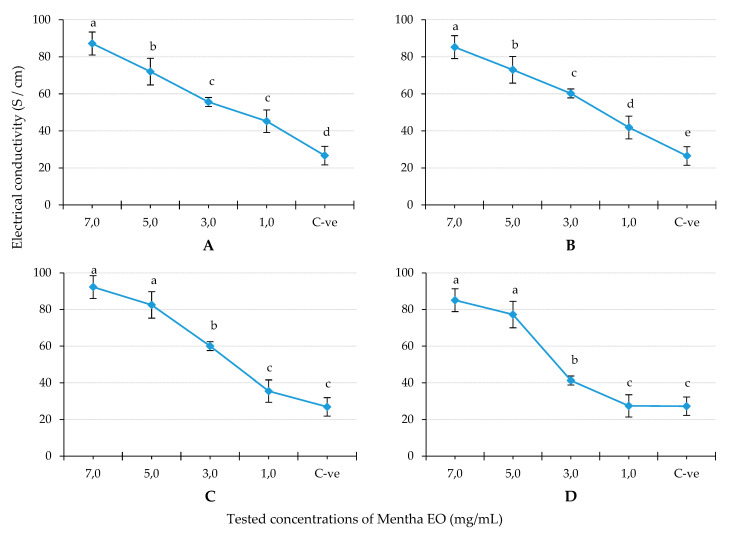
The effect of peppermint EO on mycelium electrical conductivity of the tested fungi. Where, (**A**): *Monilinia fructicola*; (**B**): *Botrytis cinerea*; (**C**): *Aspergillus niger*; (**D**): *Penicillium expansum*. C-ve: negative control (potato dextrose broth). Differences between the tested concentrations for each tested fungus indicate mean values significantly different at *p* < 0.05 according to one-way ANOVA for each fungus combined with Duncan post hoc multiple comparison test.

Figure 2 showed the effect of peppermint EO at different doses on the mycelium electrical conductivity (MEC) as indication of the cell membrane permeability (CMP) of the four tested fungi. Generally, the effect of the studied EO on the CMP of all tested fungi was dose-dependent. In particular, the highest tested concentration (7.0 mg/mL) showed the EC values 87.2, 85.3, 92.3 and 85.1 S/cm corresponding to the CMP of *M. fructicola*, *B. cinerea*, *A. niger* and *P. expansum*, respectively. On the other hand, the concentration 7.0 mg/mL showed a significant increase in the CMP in the case of *M. fructicola* and *B. cinerea*, whereas there was no significant difference between the two doses 5.0 and 7.0 mg/mL regarding *A. niger* and *P. expansum.* In addition, there was a dramatical increase in the CMP in the case of *P. expansum* after treatment with 5.0 mg/mL.

**Figure 3:**
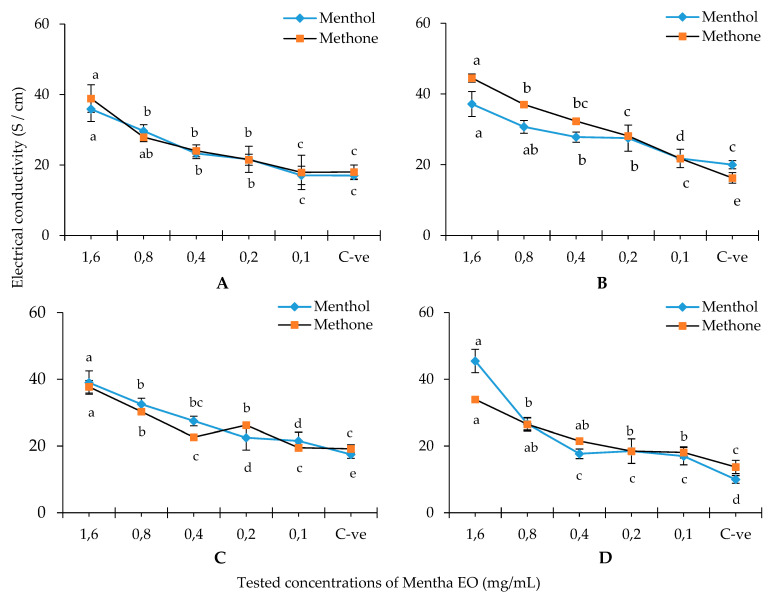
The effect of single constituents of peppermint EO on mycelium electrical conductivity of the tested fungi. Where (**A**): *Monilinia fructicola*; (**B**): *Botrytis cinerea*; (**C**): *Aspergillus niger*; (**D**): *Penicillium expansum*. C-ve: negative control (potato dextrose broth). Differences between the tested concentrations for each tested fungus indicate mean values significantly different at *p* < 0.05 according to one-way ANOVA for each fungus combined with Duncan post hoc multiple comparison test.

Figure 3 showed the effects of two single constituents of peppermint EO (menthol and menthone) at different doses on the MEC of the four tested fungi. In the case of menthol, it showed a dose-dependent effect on CMP of *M. fructicola*, *B. cinerea* and *A. niger,* whereas the CMP was highly decreased after treatment with 0.8 mg/mL in the case of *P. expansum*. The EC values were 35.9, 37.2, 39.0 and 45.5 S/cm for *M. fructicola*, *B. cinerea*, *A. niger* and *P. expansum*, respectively. Regarding the menthone, it showed a dose-dependent effect on the CMP against *M. fructicola*, *B. cinerea* and *P. expansum*. The EC values were 38.9, 44.5, 37.8 and 34.0 S/cm for *M. fructicola*, *B. cinerea*, *A. niger* and *P. expansum*, respectively. 

### 2.5. Fungicidal Microdilution Broth Assay (96-Microplate)

This assay was carried out to determine the minimum fungicidal concentration (MFC) which is defined as the lowest concentration of the tested antimicrobial agent that can inhibit the growth of fungi significantly differently to the growth of the negative control, as reported by Arikan [23]. The results of the fungicidal effect of mint EO and its main single constituents on mycelium growth percentage are reported in Table 3, whereas the MFC values of peppermint EO and its two main constituents are reported in Table 4 using the tendency-line formula of the chart in Microsoft Excel. The studied EO showed 4.78, 2.91, 5.40 and 4.98 mg/mL, corresponding to the inhibition of 50% visible growth of fungal mycelium of *M. fructicola*, *B. cinerea*, *A. niger* and *P. expansum*, respectively.

Regarding menthol, the MFC values were 0.85, 1.40, 1.45 and 1.21 mg/mL against *M. fructicola*, *B. cinerea*, *A. niger* and *P. expansum*, respectively. In the case of menthone, the MFC values were 1.31, 1.37, 1.90 and 1.69 mg/mL, against *M. fructicola*, *B. cinerea*, *A. niger* and *P. expansum*, respectively. 

## 3. Discussion

The studied EO in the current study was hydrodistilled from the new cultivar “Kristinka” of *M. piperita*. The parameters of EO differ from the standard ones for *M. piperita.* The cultivar Kristinka was bred and certificated to obtain a higher amount of the main component, menthol [24]. The newly bred cultivar of *M.* × *piperita* is characterised by a higher amount of menthol than found in other commercial cultivars [20,25]. The amount of EO depends on external factors influencing the vegetation season (environmental and climatic conditions) and may vary [25,26]. Plant biodiversity is also represented by different amounts of the main chemical components. This variation present great opportunity for the research to study the different effects of antibacterial activity.

Generally, the explanation of components of EO analysed by the GCMS is by peak area and it is explained in %. In particular, the major components of the EO of *M.* × *piperita* in different publications are menthol, menthone, menthofuran and menthyl acetate in the amounts of about 40, 30, 7 and 10%, respectively, of the whole amount of EO content [2,7,27,28,29]. Another study conducted by Kamatou et al. [18] on *Mentha canadensi* EO reported that the main components were identified as isomenthone (27.4%), menthol (24.3%), menthone (9.2%), limonene (5.8%), 1,8-cineole (5.6%), menthofuran (4.4%) and isomenthol (3.2%).

Many studies have highlighted the promising antibacterial and antifungal activity of peppermint EO against some human- and phytopathogens such as *Botrytis cinerea*, *Cladosporium cladosporioides*, *Penicillium aurantiogriseum*, *Staphylococcus aureus*, *Streptococcus pyogenes*, *Escherchia coli* and *Klebsiella pneumonia* [2,7,27]. On the other hand, the antimicrobial activity of peppermint EO might be correlated to its chemical composition due to the hydrophobic nature of those above-mentioned compounds, which allows them to interact with microbial membranes causing cell lysis, interrupting the proton’s motor force, electron flow and transport activity, and inhibiting protein synthesis [30,31]. Particularly, the obtained results of the current study of the bioactivity of menthol and menthone have confirmed their role in antimicrobial activity, as previously hypothesized.

Regarding the antifungal activity of peppermint EO, Tsao and Zhou [32] concluded that menthol was able to inhibit the postharvest fungi *Botrytis cinerea* and *Monilinia fructicola* [32]. Furthermore, different stereoisomers of menthol were active against *Fusarium verticillioides*, commonly reported as fungal species infecting maize (*Zea mays*) [33]. Tyagi et al. [34] have reported the efficacy of peppermint oil and its vapours against yeasts causing food spoilage in fruit juice such as *Saccharomyces cerevisiae*, *Zycosaccharomyces bailii*, *Aureobasidium pullulans*, *Candida diversa*, *Pichia fermentans*, *Pichia kluyveri*, *Pichia anomala* and *Hansenula polymorpha.*

A recent study conducted by Hsouna et al. [5] underlined the antibacterial activity of peppermint EO against *Agrobacterium tumefaciens*, the causal agent of crown gall disease in over 140 species of eudicots, where the tested concentration 200 mg/mL was able to completely inhibit the formation of tumours on tomato plants when inoculated with *A. tumefaciens* ATCC 23308^T^ [5].

In the current research, peppermint EO showed promising antifungal activity against the postharvest tested pathogenic fungi by measuring the growth of mycelium in plates. In addition, the studied EO explicated moderate to acceptable antibacterial activity, especially against *P. syringae* pv. *phaseolicola* and *C. michiganensis*, by measuring the diameter of the inhibition zones compared to the respective positive controls.

The results of the inhibitory effect against some phytopathogens are in agreement with some other important studies, especially those conducted by Afridi et al. [35]. The latter authors have attributed the biological activity of peppermint EO to their ability to penetrate the plasma membranes and cell walls of fungal cells, increasing their permeability, causing a significant decomposition of the walls, and later leading to the death of the fungal cells [32]. The last interpretation is what we tried to clarify through this research by conducting the CMP assays of cell membranes and their rate of electrical conductivity in the broth culture media of the tested fungi [36,37]. This, in turn, gave a clear indication of a change in the normal rate of permeability of the cell wall compared to the control cells due to the influence of biological oil as well as mono active compounds.

Consistently with this interpretive context, Ultee et al. [38] have also attributed the promising biological activity of peppermint EO to its rich content of menthol and its related compounds. These compounds can destabilize the cytoplasmic membrane and act as a proton exchanger to reduce pH gradient. Therefore, this action can destroy the proton motive force cause the depletion of ATP and hence increasing the possibility of cell death.

## 4. Materials and Methods

### 4.1. Plant Materials and Extraction of Essential Oil

Peppermint plant was grown in the experimental field belonging to University of Prešov in 2020. It was harvested in the flowering developmental stage, then dried in the shade for several days. When the plant materials were able to be crushed in the hands, they were placed into the Clevenger apparatus for hydrodistillation of EO. Fifty grams of plant materials were placed into glass flask, covered with water and connected to the Clevenger apparatus. After 3 h of hydrodistillation, the EO was reached. The procedures were repeated a few times to obtain amount of EO necessary for the successive chemical and biological analysis. Quantitative parameter was calculated as an average amount of all hydrodistillation, recalculated as a percentage of amount of utilized plant materials.

### 4.2. Gas Chromatography-Mass Spectrometry Analysis

Three samples of peppermint EO were analysed by a gas chromatography/mass spectrometry (GC/MS) for qualitative properties in laboratory at University of Prešov. GC/MS analyses were carried out on devices Varian 450-GC and 220-MS. Separation was conducted on a capillary column BR 5ms (30 m × 0.25 mm ID, 0.25 μm film thickness). Injector type 1177 was heated to a temperature of 220 °C. Injection mode was splitless (1 μL of a 1:1000 n-hexane solution). Helium was used as a carrier gas at a constant column flow rate of 1.2 mL min^−1^. Column temperature was programmed as follows: initial temperature was 50 °C for 10 min, then increased to 100 °C for 3 min, maintained isothermally for 5 min and then increased to 150 °C for last 10 min. The total time for analysis was 46.67 min. The MS trap was heated to 200 °C, manifold 50 °C and transfer line 270 °C. Mass spectra were scanned every 1 s in the range 40–650 *m*/*z*. The retention indices were determined in relation to the Rt values of a homologous series of n-alkanes (C10–C35) under the same operation conditions. Constituents were identified by comparison of their retention indices (RI) with published data in different literature. Further identification was made by comparison of the mass spectra with those stored either in NIST 02 library or with those from the literature [39]. Components relative concentrations were obtained by percentage of peak area normalization.

### 4.3. Preliminary Screening of Antimicrobial Activity

#### 4.3.1. Tested Bacterial and Fungal Isolates

The tested bacterial strains are: *Clavibacter michiganensis* Smith, and *Xanthomonas campestris* Pammel, *Pseudomonas savastanoi* Janse (Gardan) and *P. syringae* pv. *phaseolicola* Van Hall were used in this assay, whereas four postharvest phytopathogenic fungi: *Monilinia fructicola* (G. Winter) Honey, *Aspergillus niger* van Tieghem, *Penicillium expansum* Link, and *Botrytis cinerea* Pers were used for the antifungal activity assay. All tested isolates were identified by classical and molecular methods and conserved as pure culture in the collection of the School of Agricultural, Forestry, Food and Environmental Sciences (SAFE), University of Basilicata, Potenza, Italy.

#### 4.3.2. Antibacterial Activity

Disc diffusion assay. The antibacterial test was carried out following the disc diffusion method [40,41] using the King B nutrient media (KB) [42]. A bacterial suspension of each tested bacteria was prepared in sterile distilled water adjusted at 10^6^ CFU/mL (OD ≈ 0.2 nm) using a turbidimetry instrument (Biolog, Hayward, CA, USA). Four millilitres of bacterial suspension mixed with soft agar (0.7%) at ratio 9:1 (*v/v*) were poured over each plate (90 mm diameter). Blank discs of 6 mm (OXOID, Milan, Italy) were then placed over the KB-plate surfaces and about 20 µL from each tested EO concentration at 0.1, 1 and 10 mg/mL was carefully applied over discs. Tween 20 was added to each tested EO concentration at 0.2% for accelerating the oil solubility. Tetracycline (1.6 mg/mL) was used as a positive control. The antibacterial activity was estimated by measuring the diameter of inhibition zone in mm ± SDs around each treated disc compared to the positive control ones.

#### 4.3.3. Antifungal Activity

Incorporation assay. The possible fungicidal activity of the studied EO was evaluated at three different doses (0.1, 1 and 5 mg/mL) following the incorporation method [43,44,45] as explained below. The EO was incorporated into Potato Dextrose Agar (PDA) medium at 45 ± 2 °C. Fungal disks (0.5 cm) from each of the phytopathogenic fungi (96 h fresh culture) were deposited in the centre of each Petri dish. All plates were incubated at 22 ± 2 °C for 96 h in darkness. As negative control, PDA plates without any treatments were inoculated only with each fungus. The diameter of fungal mycelium growth was measured in mm ± Standard Deviations (SDs) between the three replicates [46] and the percentage of growth inhibition (PGI%) was calculated using Equation (1) [47] compared to synthetic fungicides Azoxystrobin (0.8 µL/mL), a large spectrum fungicide, as control according to the international limit of microbicide standards.

(1) $$\mathrm{PGI}\;\left(\%\right)=\frac{\mathrm{GC}-\mathrm{GT}}{\mathrm{GC}}\times 100$$

where PGI is the percentage of growth inhibition, GC is the average diameter of fungal mycelium in PDA negative control and GT is the average diameter of fungal mycelium on the oil-treated PDA dish.

### 4.4. Cell Membrane Permeability

The CMP effect of the mint EO and its two main principals was determined by measuring the potential of electrical current transport through water as molar conductivity (MC) or electrolytic conductivity (EC) as reported by Elshafie et al. [21]. This assay was performed by transferring five mycelial discs (0.5 cm diameter) from fresh culture of each tested fungus into Potato Dextrose Broth (PDB) medium and incubated under shaking condition (180 rpm/min), at 28 °C for 96 h. A gram and half of dried mycelia from each fungal species was re-suspended into 20 mL of each tested EO concentration at 1, 3, 5 and 7 mg/mL or single components at 0.1, 0.2, 0.4, 0.8 and 1.6 mg/mL and incubated at 22 ± 2 °C. EC values have been measured after 72 h of incubation. The IP% of EC value was calculated following Equation (2) [21].

(2) $$\mathrm{IP}\;\left(\%\right)=\frac{\mathrm{EC}\;t}{\mathrm{EC}\;\mathrm{ctrl}}\times 100$$

where EC t. is the electrical conductivity of the treated sample and EC ctrl. is the electrical conductivity of the PDB broth culture.

### 4.5. Fungicidal Microdilution Broth Assay

The MFC was carried out against the four tested pathogenic fungi using a 96-well microplate (Nunc MaxiSorp^®^, Vedbaek, Denmark) by a micro-dilution method [22,48]. Four millilitres of liquid suspension from fresh fungal cultures (96 h) was prepared at 10^8^ spore/mL. The tested EO was dissolved in PDB at 1.0, 3.0, 5.0 and 7.0 mg/mL, whereas the tested concentrations for menthol and menthone were 0.1, 0.2, 0.4, 0.8 and 1.6 mg/mL. The proposed concentrations of this assay were selected according to the obtained results from the preliminary in vitro antifungal assay. One hundred microlitres/well from each prepared concentration of EO and 100 µL/well of PDB media were added into the 96-well microplates then 30 µL/well of fungal suspension from each tested fungus was uploaded per all wells. All plates were incubated at 24 ± 2 °C. The absorbance was measured at λ = 450 nm using microplate reader instrument (DAS s.r.l., Rome, Italy) after 24 h. The MGP percentage was calculated using Equation (3). The whole experiment was repeated in triplicate ± SDs.

(3) $$\mathrm{MGI}\;\left(\%\right)=\frac{\mathrm{Abs}.\;t}{\mathrm{Abs}.\;c}\times 100$$

where Abs. t: is the value of the absorbance at 450 nm for each treatment; Abs. c: is the value of the absorbance at 450 nm for the PDA + fungi as control.

### 4.6. Statistical Analysis

The obtained results of the biological assays were subjected to one-way ANOVA for the statistical analysis. Then, the significance level of the findings was checked by applying *Tukey* B Post Hoc multiple comparison test with a probability of *p* < 0.05 using statistical Package for the Social Sciences (SPSS) version 13.0 (Prentice Hall: Chicago, IL, USA, 2004).

## 5. Conclusions

The EO from *Mentha* × *piperita* cv. ‘Kristinka’ demonstrated promising antifungal activity against some serious phytopathogenic fungi even at low concentrations and this result is very interesting, especially for controlling postharvest fungi. In addition, the studied EO showed acceptable antibacterial activity against the following pathogenic bacteria: *P. syringae* pv. *phaseolicola* and *C. michiganensis*; however, it showed little effect against *P. savastanoi,* and no activity against *X. campestris*. On the other hand, the biological activity of the studied EO can be highly attributed to its rich content of menthol (70.08%) and menthone (14.49%). The MFC value of the fungicidal effect achieved by the peppermint EO was 2.9 mg/mL against *B. cinerea* and the MFC value in the case of menthol was 0.8 mg/mL against *M. fructicola*, whereas menthone achieved MFC values equal to 1.3 mg/mL against *M. fructicola* and *B. cinerea*.

The obtained promising results of the antimicrobial activity of peppermint EO proved its potential to control several fungal and bacterial pathogens. Furthermore, the studied EO and its two main constituents can be used successfully in the chemical industries as natural alternatives to synthetic substances against several phytopathogens.

## Figures and Tables

**Table 1:** Identification of *Mentha* × *piperita* cv. ‘Kristinka’ EO components.

Components	*Mentha* × *piperita* cv. ‘Kristinka’				
	Ki Exp	Ki Lit.	%	Formula	Chem. Group
α-Pinene	935	936	0.47 ± 0.01	C_10_H_16_	MH
β-Pinene	976	978	0.45 ± 0.01	C_10_H_16_	MH
Limonene	1025	1025	4.32 ± 0.03	C_10_H_16_	MH
cis-β-Ocimene	1028	1029	0.02 ± 0.00	C_10_H_16_	MH
Menthone	1146	1136	14.49 ± 0.01	C_10_H_18_O	OM
Menthol	1174	1172	70.08 ± 0.05	C_10_H_20_O	OM
α-Terpineol	1177	1176	0.18 ± 0.00	C_10_H_18_O	OM
Carvone	1210	1214	0.01 ± 0.00	C_10_H_14_O	OM
Piperitone	1223	1226	0.60 ± 0.02	C_10_H_16_O	OM
Isopulegol acetate	1271	1263	0.01 ± 0.00	C_12_H_20_O_2_	OM
Menthyl acetate	1278	1280	3.76 ± 0.01	C_12_H_22_O_2_	OM
α-Cubebene	1354	1355	0.01 ± 0.00	C_15_H_24_	SH
Clovene	1365	1365	0.03 ± 0.00	C_15_H_24_	SH
Isoledene	1370	1382	0.01 ± 0.00	C_15_H_24_	SH
β-Bourbonene	1386	1378	0.07 ± 0.01	C_15_H_24_	SH
β-Elemene	1389	1389	0.03 ± 0.00	C_15_H_24_	SH
β-Cubebene	1390	1390	0.17 ± 0.02	C_15_H_24_	SH
Longifolene	1404	1411	0.04 ± 0.00	C_15_H_24_	SH
α-Gurjunene	1410	1413	0.20 ± 0.01	C_15_H_24_	SH
(Z)-β-Farnesene	1420	1420	0.50 ± 0.00	C_15_H_24_	SH
β-Caryophyllene	1421	1421	2.96 ± 0.04	C_15_H_24_	SH
Aristolene	1422	1423	0.02 ± 0.00	C_15_H_24_	SH
Aromadendrene	1435	1443	0.01 ± 0.00	C_15_H_24_	SH
α-Humulene	1448	1455	0.07 ± 0.00	C_15_H_24_	SH
Allo-Aromadendrene	1460	1462	0.20 ± 0.01	C_15_H_24_	SH
γ-Gurjunene	1472	1472	0.14 ± 0.01	C_15_H_24_	SH
β-Chamigrene	1474	1474	0.03 ± 0.00	C_15_H_24_	SH
γ-Muurolene	1475	1474	0.01 ± 0.00	C_15_H_24_	SH
α-Amorphene	1477	1477	0.03 ± 0.00	C_15_H_24_	SH
Germacrene D	1479	1479	0.03 ± 0.00	C_15_H_24_	SH
Ledene	1489	1491	0.53 ± 0.02	C_15_H_24_	SH
Valencene	1493	1494	0.06 ± 0.00	C_15_H_24_	SH
α-Muurolene	1496	1496	0.06 ± 0.01	C_15_H_24_	SH
γ-Cadinene	1507	1507	0.09 ± 0.01	C_15_H_24_	SH
δ-Cadinene	1520	1520	0.16 ± 0.02	C_15_H_24_	SH
Spathulenol	1565	1572	0.03 ± 0.00	C_15_H_24_O	OS
Total			99.88		

Percentage was calculated as an average from three replication ± SD; Ki–Kovats index calculated by researchers; Ki lit. Kovats index from literature (MS Finder) for comparison.

**Table 2:** Antibacterial activity of *Mentha* × *piperita* cv. ‘Kristinka’ EO.

Tested EOs	Diameter of Inhibition Zones (mm)				
	Conc.	*X. campestris*	*C. michiganensis*	*P. syr.* pv. *phaseolicola*	*P. savastanoi*
Peppermint EO	10 mg/mL	0.0 ± 0.0 b	27.5 ± 2.8 b	39.5 ± 0.5 a	09.0 ± 1.2 b
	1 mg/mL	0.0 ± 0.0 b	17.0 ± 2.3 c	26.5 ± 1.6 b	0.0 ± 0.0 c
	0.1 mg/mL	0.0 ± 0.0 b	09.0 ± 1.1 d	19.5 ± 0.6 c	0.0 ± 0.0 c
Tetracycline (1.6 mg/mL)		23.5 ± 1.70 a	39.5 ± 0.6 a	40.0 ± 1.6 a	37.0 ± 2.2 a

Values followed by different letters for each tested bacterium in each column are significantly different at *p* < 0.05 according to two-way ANOVA combined with Duncan post hoc test. Data are expressed as the mean of three replicates ± SD.

**Table 3:** Fungicidal effect of EO and single constituents on mycelium growth (%) in broth culture.

Tested Concentrations (mg/mL)	Mycelium Growth Percentage (MGP)			
	*M. fructicola*	*B. cinerea*	*A. niger*	*P. expansum*
*M. piperita* 7.0	33.3 ± 4.0 d	31.6 ± 6.0 d	35.1 ± 4.1 d	18.6 ± 3.0 c
*M. piperita* 5.0	47.8 ± 4.0 c	42.8 ± 2.4 bc	71.3 ± 3.4 c *	48.6 ± 4.0 b
*M. piperita* 3.0	60.5 ± 6.0 c *	48.9 ± 0.6 bc	74.3 ± 0.5 ab	50.1 ± 3.0 b
*M. piperita* 1.0	77.8 ± 4.1 ab	66.1 ± 5.9 b *	78.6 ± 1.9 ab	52.4 ± 0.5 b *
C-ve: PDB + F	100.0 ± 0.0 a	100.0 ± 0.0 a	100.0 ± 0.0 a	100.0 ± 0.0 a
Principal Single Components				
Menthol 1.6	40.0 ± 5.6 c	41.7 ± 4.3 d	58.1 ± 6.2 c	51.9 ± 7.4 cd
Menthol 0.8	50.5 ± 6.6 c	66.1 ± 5.7 c *	60.0 ± 5.8 c	52.8 ± 7.3 cd
Menthol 0.4	60.1 ± 4.3 b	78.2 ± 1.3 ab	62.1 ± 4.4 c	66.4 ± 5.0 c
Menthol 0.2	65.0 ± 5.0 b *	78.7 ± 3.3 ab	71.2 ± 3.8 b *	76.7 ± 5.3 b *
Menthol 0.1	69.1 ± 2.9 ab	81.4 ± 6.3 ab	93.8 ± 3.5 a	85.2 ± 4.2 ab
Menthone 1.6	29.2 ± 4.4 d	42.9 ± 2.0 d	45.1 ± 3.0 d	33.6 ± 3.3 d
Menthone 0.8	55.8 ± 4.0 c	66.0 ± 3.7 c	63.5 ± 4.5 c	60.6 ± 6.7 c
Menthone 0.4	64.2 ± 6.2 b *	70.4 ± 3.4 b *	71.3 ± 3.3 b	68.9 ± 5.8 b *
Menthone 0.2	69.6 ± 2.2 ab	89.2 ± 4.7 a	76.6 ± 4.2 b	79.2 ± 4.3 ab
Menthone 0.1	75.7 ± 1.5 ab	97.1 ± 0.6 a	77.0 ± 4.2 b *	83.2 ± 2.3 ab
C-ve: PDB + F	100.0 ± 0.0 a	100.0 ± 0.0 a	100.0 ± 0.0 a	100.0 ± 0.0 a

Values followed by different letters in each column for each tested fungi are significantly different at *p* < 0.05 according to one-way ANOVA combined with *Tukey* B post hoc test. (*) are the mycelium growth percentages corresponding to the MFC values. Data are expressed as the mean of three replicates ± SD and presented for peppermint EO and the two single substances.

**Table 4:** MFC values of fungicidal effect of studied EO and single constituents.

MFC (mg/mL)				
	*M. fructicola*	*B. cinerea*	*A. niger*	*P. expansum*
*M. piperita* EO	4.78	2.91	5.40	4.98
Menthol	0.85	1.40	1.45	1.21
Menthone	1.31	1.37	1.90	1.69

## Data Availability

Not applicable.
